# Primary Care Physicians’ Beliefs and Practices Regarding E-Cigarette Use by Patients Who Smoke: A Qualitative Assessment

**DOI:** 10.3390/ijerph13050445

**Published:** 2016-04-26

**Authors:** Omar El-Shahawy, Richard Brown, Jennifer Elston Lafata

**Affiliations:** 1 Section on Tobacco, Alcohol and Drug Use, Department of Population Health, School of Medicine, New York University, New York, NY 10016, USA; 2 Public Health Research Center, New York University Abu Dhabi, Abu Dhabi 129188, UAE; 3 Department of Health Behavior and Policy, School of Medicine and Massey Cancer Center, Virginia Commonwealth University; Richmond, VA 23284, USA; richard.brown@vcuhealth.org (R.B.); jennifer.elston-lafata@vcuhealth.org (J.E.L.)

**Keywords:** primary care, decision making, smoking cessation, harm reduction, e-cigarettes

## Abstract

We explored primary care physicians’ (PCPs’) beliefs and practices about e-cigarettes. Cross-sectional, semi-structured interviews with PCPs in 2014 were conducted and audio-recorded. Participants were 15 general internal and family medicine physicians practicing in two settings in Virginia, USA. Interview recordings were transcribed, and the content analyzed using the Constant Comparative Method to identify key themes regarding PCPs’ reported current practices and beliefs. Five themes were identified: (1) existing clinic processes do not include mechanisms to screen for noncombustible tobacco products (such as e-cigarettes); (2) e-cigarette discussions are becoming commonplace with patients initiating the discussions and seeking physician guidance regarding e-cigarette use; (3) a lack of knowledge regarding the potential harms and benefits of e-cigarettes, yet a willingness to support their patients’ desire to use e-cigarettes (4) believing e-cigarettes are a safer alternative to smoking combustible tobacco products; and (5) abandoning concerns regarding the potential harms of e-cigarettes in the context of highly addicted patients and those with extensive comorbidities. Despite acknowledging limited knowledge regarding e-cigarettes, findings suggest that some PCPs are currently recommending e-cigarettes to their patients for smoking cessation and relative harm reduction, often personalizing recommendations based on the patient’s perceived addiction level and current health status. Physicians need to be informed about the evolving evidence regarding the risks and benefits of e-cigarettes.

## 1. Introduction

A wide range of new and emerging tobacco products are thriving in the United States (U.S.) despite limited knowledge of their health implications [1,2]. One such product, the e-cigarette, is marketed as a cessation aid, harm reduction strategy, or both [2,3]. As evidenced by a number of recent studies, experimentation, use, and promotion of e-cigarettes via conventional as well as online marketing and social media channels have been growing exponentially over the past few years [1,4,5,6,7,8,9]. Further, physician endorsements are being used in e-cigarette advertising which is reminiscent of combustible cigarette advertising in the 1950s [10,11]. However, despite growing e-cigarette use, how they are perceived by physicians is not fully understood [12,13].

Prior reports indicate that users of e-cigarettes perceive a diverse array of benefits from their use, ranging from effectiveness in cessation assistance [14] to improved taste and smell [15]. Nevertheless, there are also reports citing adverse events resulting from e-cigarette use [16]. Whether physicians are knowledgeable of such adverse events is unknown. Prior reports suggest that physicians are turning to media and their patients for e-cigarette-related information, among other sources [12]. Since the current tobacco use counseling guidelines do not address e-cigarettes [17], understanding what guides physicians’ practice when recommending e-cigarette use is of ample importance.

National medical organizations such as the American Heart Association [18], American Association for Cancer Research and the American Society of Clinical Oncology [19] have recently issued policy statements regarding e-cigarettes. Such organizations have advocated that physicians continue to recommend only Food and Drug Administration (FDA)-approved pharmacotherapies for cessation. Most recently, the U.S. Preventive Services Task Force continued to support the use of only FDA-approved pharmacotherapies for cessation and not e-cigarettes, citing the lack of sufficient evidence surrounding e-cigarette potential to aid with smoking cessation [20].

With the absence of either a comprehensive set of recommendations from professional organizations or regulatory actions from the FDA [21], physicians are likely to rely on their own beliefs and perceptions when discussing e-cigarette use with their patients who smoke. Current evidence suggests that e-cigarettes are being discussed with primary care physicians (PCPs) as well as specialty physicians such as oncologists, cardiologists and pulmonologists [12,13,22,23]. Yet, to our knowledge, there are only two examples of published reports that include U.S.-based PCPs [13,23]. Both reports rely solely on data from physician surveys, and found that patients actively solicit their PCP’s opinions regarding e-cigarettes. Despite these studies, the manner in which PCPs approach e-cigarette discussions, and the full range of factors that contribute to their beliefs, perceptions and decisions to recommend e-cigarettes remain largely unknown [12,13].

Guided by the theoretical tenants of the Theory of Reasoned Action [24], the purpose of this study is to describe the range of tobacco and nicotine products included in PCPs’ current tobacco use screening behavior. We also sought to explore their beliefs and practices regarding e-cigarette use, and to understand the context in which they might recommend e-cigarette use to their patients who smoke.

## 2. Materials and Methods

### 2.1. Study Participants

Participants were family and general internal medicine physicians employed by a large university health system in Richmond, Virginia, supplemented by an additional sample of family medicine physicians practicing in the Virginia Ambulatory Care Outcomes Research Network (ACORN) located in northern Virginia. ACORN is a network of small to medium primary care practices that are not university-based but are committed to improving health and transforming care delivery through practice based research [25]. We purposefully sampled from different practice settings to ensure that sampled physicians treated heterogeneous patient populations across a diversity of settings and geographic areas in Virginia. In April of 2014, we contacted all family and general internal medicine physicians working at the university health system (*n* = 46) via e-mail to invite them to participate in the study. In July 2014, we invited another group of (*n* = 26) family medicine physicians practicing in two ACORN clinics. To be eligible for participation, physicians had to report providing outpatient primary care to adult patients and discussing tobacco use with at least one of their patients within the past 30 days. Participants did not receive any compensation for participation. All subjects gave their informed consent for inclusion before they participated in the study. The study was conducted in accordance with the Declaration of Helsinki, and the protocol was approved by the Ethics Committee of Virginia Commonwealth University (HM20000547_CR3).

### 2.2. Data Collection

After providing written informed consent, demographic and practice information (*i.e.*, age, gender, race/ethnicity, primary specialty, weekly patient volume and year of training completion) was collected from each participant. Each PCP then participated in an in-depth, semi-structured interview. The interview guide was designed to elicit (a) current tobacco use screening and counselling practices, (b) perceptions of and beliefs regarding e-cigarettes and (c) screening and counselling practices surrounding e-cigarette use. For the current analyses we focused on responses to nine questions (See Table 1). All interviews were conducted in person by the study PI (Omar El-Shahawy) between April and August, 2014 at the PCPs’ offices. Interviews were audio-recorded, and ranged between 23 and 55 minutes. Prior to analyses all interviews were transcribed verbatim.

### 2.3. Coding and Analytic Methods

Prior to coding, names and other identifying information were removed from transcripts. Transcripts of audio-recorded interviews were analyzed using the Constant Comparative Method proposed by Glaser [26]. The research team (Omar El-Shahawy, Richard Brown, and Jennifer Elston Lafata) conducted bi-weekly meetings during which themes were identified and discussed. A consensus process was used to achieve agreement on the inclusion of themes. Initially, as the methodology requires, a first set of five transcripts was analyzed. Once an exhaustive analysis of this original data set was complete, a second set of five transcripts was analyzed. The themes which emerged from these data were compared with those from the original data set and if necessary, new themes were added. Few new themes emerged; however, to comply with the Constant Comparative Method, we analyzed the remaining five transcripts to ensure theoretical saturation. As no new themes emerged after this process we concluded that we had reached saturation. This iterative process yielded further refinement of the names of the five themes.

## 3. Results

### 3.1. Study Population

Fifteen PCPs, seven from the university health system and eight from ACORN consented to participate in the study. Eleven participants were family medicine physicians and four were general internal medicine physicians. The mean age of participants was 43.1 years (SD = +10.3) and on average they had been practicing for 15.4 years (SD = +10.6). PCPs were evenly distributed by gender (*i.e*., 53% male and 47% female), and were predominantly white (60%). The average patient volume was 63.2 patients per week (SD = +31.9).

### 3.2. Themes

Thirteen PCPs reported that they have previously talked about e-cigarettes with their patients. When probing as to whether those e-cigarette related discussions resulted in any prior recommendations, six of those 13 PCPs reported having previously recommended e-cigarette use to at least one of their patients. Five overarching themes emerged from the qualitative analysis: (1) PCPs acknowledge that existing clinic processes do not include mechanisms to screen for noncombustible tobacco products (such as e-cigarettes), (2) PCPs report that e-cigarette discussions are becoming commonplace in practice with patients initiating the discussions and seeking physician guidance regarding e-cigarette use, (3) PCPs express a lack of knowledge regarding the potential harms and benefits of e-cigarettes, yet a willingness to support their patients’ desire to use e-cigarettes (4) PCPs believe that e-cigarette use is a safer alternative to smoking combustible tobacco products, and (5) PCPs’ concerns regarding the potential harms of e-cigarettes are abandoned in highly addicted patients and those with extensive comorbidities. Each theme is described below with illustrative interpolations from transcript data.

#### 3.2.1. Theme 1: PCPs Acknowledge that Existing Clinic Processes Do Not Include Mechanisms to Screen for Noncombustible Tobacco Products (such as E-Cigarettes)

Given the emergence of noncombustible tobacco products, such as e-cigarettes, it could be expected that clinic-based tobacco screening processes are emerging to encompass noncombustible products in parallel. However, when asked about their screening for tobacco use, PCPs reported no such process for addressing noncombustible tobacco products.

Even when specifically asked whether they have ever asked a patient about their use of e-cigarettes, PCPs indicated that neither they nor their clinic staff probe about the use of noncombustible tobacco products. For example, one physician responded: *“Typically we’ll ask as part of the routine screening, but I will admit that for most routine visits, I generally don’t probe into smokeless tobacco products”* (PCP A). Likewise, another PCP said: *“I don’t ask specifically about smokeless tobacco, chewable tobacco, e-cigarettes. It’s generally just ‘Do you smoke?’ or ‘Were you a smoker in the past?’ and then ‘How much, over what period of time?”* (PCP B). Other existing clinic processes were likewise not reported to include consideration of e-cigarettes. One PCP illustrated this when asked about the process of tobacco use screening in their clinic by saying: “*So we actually primarily, when the nurses ask the patients, they ask them whether they smoke… and then on our intake sheet for the patients, it does ask them about any tobacco use and then a lot of people, I think, only think about smoking when they read the question, but some people do put things on there, like tobacco chew. I don’t think too many people put, you know, like e-cigarettes or anything like that on there. They just kind of put information about tobacco, cigarettes really*” (PCP L). As illustrated by these quotes, established clinic processes do not appear to be evolving to include the emergence of new and modified tobacco products.

#### 3.2.2. Theme 2: PCPs Report that E-cigarette Discussions Are Becoming Commonplace in Practice with Patients Initiating the Discussions and Seeking Physician Guidance regarding E-Cigarette Use

Although PCPs acknowledge a void in clinic processes to screen for and record the use of noncombustible tobacco products, they report that e-cigarette discussions are becoming commonplace during primary care office visits. For example, when asked “Have any of your patients ever asked you about e-cigarettes?”, a PCP responded: “*E-cigarettes have definitely been coming up in the last six months. I would say maybe the last year, but in the last six months more and more patients are mentioning it as an alternative or something they are looking to instead of traditional smoking”* (PCP E). Another example of a potentially increasing trend of e-cigarette discussions in physicians’ offices is when PCP A was asked “Have any of your patients ever asked you about e-cigarettes?”, and responded: “*They did, a lot. I mean recently. So you know usually by Question 2 or 3, you know if I get somebody who says ‘Yeah, I’ve thought about it,’ I’m usually not the one to bring it up ‘cause I’m still trying I think to figure out how to frame that. Where it’s come up probably the most in the last 12 months or so as they become more aggressive and marketed is I’ll get asked that question.*” Even more, another physician said: *“E-cigarettes come up all the time now, sometimes our patients have started doing them on their own, or they have friends who are doing them and they ask about them, so they come up pretty routinely now”* (PCP D). 

Further, PCPs report being asked for guidance about e-cigarettes, which was reflected in prior quotes, but one more PCP elaborated: *“I’ve had one of two paths sort of happened. So one is patients will ask me about e-cigarettes and are they safe and are they better than regular cigarettes. So that’s one line of questions that patients will bring up. The other thing that will happen, the other way the conversation will go is, if they’re using e-cigarettes, or my patients who said ‘I switched from smoking to e-cigarettes,’ So those are the two paths that I will have with conversations with patients”* (PCP F). As illustrated by these quotes, regardless of a void in clinic processes to identify e-cigarette use, PCPs report having conversations with their patients about e-cigarettes, and specifically, having patients ask them for advice regarding the use of e-cigarettes which seemed for them to be increasing by time.

#### 3.2.3. Theme 3: PCPs Express a Lack of Knowledge Regarding the Potential Harms and Benefits of E-Cigarettes, yet a Willingness to Support Their Patients’ Desire to Use E-Cigarettes

Physicians expressed a lack of empirical evidence regarding the potential harms and possible benefits of e-cigarettes. All PCPs, regardless of whether they had recommended e-cigarettes expressed a lack of knowledge about e-cigarette safety and their efficacy as a smoking cessation aid. One PCP who had not recommended e-cigarette use said*:* “*The safety is not listed there and you don’t know what they’re actually putting into it. They may not be labeling it correctly and that you may be putting other carcinogens in yourself and maybe you’re not getting as much smoke, but there are other things that you’re getting”* (PCP H).

With regard to the efficacy of e-cigarettes for smoking cessation, one PCP commented on the need for scientific evidence and commented that such evidence regarding e-cigarettes is lagging behind that of other established FDA-approved pharmacotherapies by saying*: “I want to see a research study that shows that that’s helped. There are great research studies with Chantix, with Wellbutrin, with patches and with doctors’ counseling. So we know that patients on average, 7% of patients quit smoking just on their own volition. If you start adding things like Chantix and Wellbutrin, you can get it up to 15 to 23%. I want to see a study like that, that randomizes people to e-cigarettes versus Chantix, versus patches, versus doctors just telling people to quit smoking, and when I see that, then I’ll say it’s an effective means of helping people quit, but there’s no data on that. It has to be studied”* (PCP F). Another PCP expressed this lack of scientific evidence as well as his willingness to support a patient trying e-cigarettes*: “I tell them is that we don’t have a lot of data on the e-cigarettes because they’re not FDA-regulated yet and so individual safety data is complicated. The only stuff I’ve been able to find is from the manufacturers and some Australian stuff, and of course that’s all done by the people that sell the cigarettes. So, I just give them all the information that we have, which is not much, and if they want to try it, I say I don’t really have a strong objection to you doing that”* (PCP D); while a third PCP reported that a patient’s expression of interest was a primary reason for the PCP to support the use of e-cigarettes for smoking cessation: “*I believe in patient-centered care, and I think that changing your health behaviors is really hard. So whatever my patient thinks is going to help them with quitting smoking, I would support, and that would include e-cigarettes, if they wanted to do that”* (PCP F). The salience of patients’ interest in trying e-cigarettes was common across all PCPs, both those who recommended e-cigarettes to their patients*: “If they bring it up and they have a motivation I’m usually very encouraging”* (PCP A), and those who had not previously recommended e-cigarette use to their patients prior to the study*: “Somebody who comes to me and specifically says, I am thinking of switching then the patient preference would be a factor in this case”* (PCP G).

As illustrated by these quotes, despite perceived voids in scientific evidence regarding either the safety of e-cigarettes or their efficacy as a cessation aid, PCPs are willing to support their patients’ use of them.

#### 3.2.4. Theme 4: PCPs Believe that E-Cigarette Use is a Safer Alternative to Smoking Combustible Tobacco Products

While PCPs question the safety of e-cigarettes, they believed them to be safer than other tobacco containing products, especially combustible cigarettes. All PCPs expressed concerns about the potential harms of e-cigarette use. However, PCPs also expressed that e-cigarette use is likely safer than the use of traditional tobacco products. As illustrated by what one PCP said when asked about e-cigarettes perceived safety*:* “*I think, on general, taken as a whole, they’re safer than smoking, chewing tobacco, pipes, cigars probably”* (PCP I). PCPs repeatedly reported using cigarette smoking as the benchmark for establishing a comparison for e-cigarettes’ safety as a nicotine delivery product. One PCP elaborated on this by saying*: “What I want to know is that they are safer than cigarettes, because it’s that risk-benefit thing. So if someone’s already smoking cigarettes, if I can’t get to perfect, which is nothing, and there are some risks associated with the inhaled nicotine, but it’s less than the inhaled cigarettes, I’ll take the e-cigarettes. I can’t imagine it’s not safer than the actual cigarettes, because cigarettes are just known to be bad for you in so many ways”* (PCP D). This perception is also clear from their tobacco use counseling screening approach. For example, some of the PCPs expressed having less concern about noncombustible tobacco products in general and e-cigarettes in particular. To illustrate this point, one PCP said: “*Usually, lesser for some reason that I am worried about chewing tobacco or snuff, I don’t ever specifically ask about e-cigarettes. So, 90% of patients I ask the question “do you smoke?” and leave it at that*” (PCP C).

Additionally, PCPs acknowledged that their perception of e-cigarettes being safer than other combustible tobacco products is a factor in their recommendation. One PCP explicitly explained this by saying*: “There is a perception--kind of automatic response--that it must be safer. Because it is not smoking, so it’s got to be better than smoking. And what I have tried to tell patients is that we don’t actually know that to be the case. We don’t know anything about e-cigarettes in terms of safety, we don’t know if they are harmful, we don’t know if they are not harmful, we do know smoking is harmful, so I often times let patients come to a decision that they are more comfortable with”* (PCP E). However, the same PCP further shared more skepticism about the absolute safety with e-cigarettes, while still acknowledging the likely relatively safety benefit of e-cigarettes compared to traditional cigarettes by saying*:* “*I am very, very skeptical about a lot of it. I think it’s being advertised as a safer, healthier alternative. I don’t think it is true and if it is, it won’t be safe, it will be safer and it still won’t be something that is very good for people.* *The vapor from the e-cigarettes has some of the chemicals that you find in tobacco smoke, and the liquid itself of e-cigarettes is incredibly dangerous”* (PCP E). Likewise, a PCP who reported that he had previously recommended e-cigarette use to a patient stated*:* “*I wouldn’t say it’s safe, because nicotine can make your heart rate go up, and vaso-constrict. If somebody takes the e-cig and takes 30 or 40 puffs in a row, that’s probably not good for their coronary vasculature. So I guess, in certain ways, you could have more harm to the heart than a regular cigarette, perhaps, in certain situations”* (PCP I). While PCPs remain skeptical regarding the safety of e-cigarettes, they nonetheless express beliefs that e-cigarettes are the lesser of two evils when compared to combustible cigarettes.

#### 3.2.5. Theme 5: PCPs’ Concerns Regarding the Potential Harms of E-cigarettes are Abandoned in Highly Addicted Patients and Those with Extensive Comorbidities

For those patients for whom PCPs perceive cigarette smoking to be an eminent threat, skepticism regarding the safety of e-cigarettes seems to be downplayed. For example, PCPs reported recommending e-cigarettes to heavy smokers or to patients with existing co-morbidities. This was illustrated by the following: *“The people who are smoking like a pack a day and really chimneys, I’m like you want anything that you can do that’s an action that gets in the right direction. So I usually am pretty encouraging of it in that setting”* (PCP K).

PCPs were also more inclined to recommend e-cigarettes for heavy long-term smokers who have previously tried quitting and failed with conventional cessation medications and who may be addicted to the social habit of smoking. For example, one PCP said*:* “*If somebody said to me, ‘Doc, I’ve already tried the gum. I’ve tried the patches. It didn’t work for me, and I’m not really interested in taking these antidepressant medicines that you’ve talked about with the craving. I think I’m just so hooked on the physical act of smoking that I think the e-cigarettes are going to be a better way for me to bridge to using,’ so I would probably recommend e-cigarettes”* (PCP J). Similarly, PCPs acknowledged that recommending e-cigarettes for cessation could be a good option for a cessation attempt with patients with smoking related co-morbidities*:* “*When I think of any therapy that I might recommend to someone without really feeling like it’s super well-established or that I really understand all the risks and benefits, it’s like people who stand the most to gain by using it, so people who are like long-term smokers or who I know will do really poorly with some of the medications or other options that are out there, people who I just think behaviorally would be more amenable to something like that, I guess those would be the people that I would think more of using it”* (PCP K). PCPs’ endorsement of e-cigarettes for their patients who smoke seems to exhibit some personalization and risk stratification: PCPs expressed that the likelihood of their recommending e-cigarettes to their patients who smoke is tied to the patient’s tobacco use profile, health status, and prior unsuccessful quit attempts.

## 4. Discussion

Five themes emerged in our current study and the information within these themes suggests that despite routine screening for conventional tobacco use, screening for e-cigarette use does not seem to be currently established in primary care. PCPs, nonetheless, report frequently discussing e-cigarettes with their patients who smoke. Furthermore, despite reporting a general lack of knowledge regarding the potential benefits and harms of e-cigarettes, PCPs perceive e-cigarettes to be a safer alternative to other tobacco products, particularly combustible cigarettes. Perhaps not surprising then, PCPs tend to recommend e-cigarettes as a safer alternative to smoking combustible tobacco products (*i.e*., a harm reduction approach) and to assist with smoking cessation for certain patient profiles, particularly those perceived as highly addicted to smoking, whose current health status warrants immediate action, and struggling to quit using FDA-approved pharmacotherapies (*i.e.,* those with multiple failed quit attempts). Moreover, patients’ interest in trying e-cigarettes appeared to be a particularly salient facilitator in a PCP’s decision to recommend e-cigarette use.

Faced with little empirical evidence [2,19], difficulty finding relevant risk/benefit information, and a void in professional guidelines [17], PCPs seem to be developing their own approaches to incorporating e-cigarette use into their tobacco-related counseling, including responses to frequent patient inquiries about e-cigarette use [12,13,23]. Prior research [12,13,23] has shown that PCPs in general believe that e-cigarettes are safer than traditional cigarettes. While PCPs in our study share that belief they were less consistent in acknowledging the efficacy of e-cigarettes as a smoking cessation aid. Nonetheless, PCPs in our sample reported being more willing to recommend the use of e-cigarettes to patients that they perceived as highly addicted or those with extensive smoking-related comorbidities compared to other smokers. Since such recommendations are being made despite PCPs’ overall skepticism regarding the efficacy of e-cigarettes as a smoking cessation aid, PCPs’ willingness to recommend e-cigarettes might be associated with their belief in e-cigarettes’ capacity for relative harm reduction for certain patients.

PCPs in our study seemed to adopt a patient-centered approach when communicating with their patients about e-cigarettes. PCPs report explaining available options to their patients, telling them the limited information they know about e-cigarettes, and soliciting their patients’ interest in using e-cigarette. Most likely, this solicitation of interest is coupled with patients informing PCPs of their own e-cigarette related knowledge. Previous studies indicate that this information provided by patients—which is heavily influenced by industry marketing and the lay press publications [9,12]—is likely to be positive [14,15]. Such information provision may in turn indirectly impact PCPs’ beliefs and practices [12]. 

Despite recommendations to screen and counsel patients for e-cigarette use [18,19], expecting most PCPs to proactively do this is likely unrealistic given the void in relevant evidence to help PCPs steer a conversation once patients’ use of, or interest in using, e-cigarettes is established. Instead, it appears that increasingly frequent office-based interactions regarding e-cigarettes are causing PCPs to develop non-evidence based opinions and then use those opinions in their routine tobacco use cessation counseling to address their subsequent patients’ inquiries about e-cigarettes. Despite the FDA and many researchers racing to fill the evidence void, the reality is that it will take many years before we understand the full range of public health benefits and risks associated with e-cigarettes [1,20], and thus the health and other implications of current PCP beliefs and practices regarding e-cigarettes.

## 5. Limitations

The results of our study should be interpreted in the context of several limitations. First, study data were collected between May and August of 2014. Given the rapidly evolving e-cigarette market the applicability of findings to today’s practices should be interpreted with caution. Second, PCPs interviewed were limited to those practicing within two Virginia settings and included only a small number of the potentially eligible physician subjects within those settings. In addition, there may be other important themes and factors not identified by the interview questions. Furthermore, PCPs’ opinions regarding e-cigarettes are likely influenced by their exposure to patients’ inquiries and discussions, e-cigarette marketing, and/or the policies and culture of the health care systems in which they were working, all of which could vary by geographic location. As such, care should be taken when generalizing findings to other settings and providers. Nevertheless, to our knowledge this is the first study to use qualitative research methods to comprehensively assess PCPs’ beliefs and practices regarding e-cigarettes.

## 6. Conclusions

In conclusion, PCPs expressed a lack of information about e-cigarette safety and efficacy along with skepticism about the role of e-cigarettes in tobacco control in general and in smoking cessation in particular. However, once a patient initiates a discussion with them, PCPs seem to be recommending e-cigarettes to their patients who smoke, while giving weight to patients’ preference in using e-cigarettes, for both smoking cessation and as a harm reduction strategy. Such findings serve to illustrate the importance of generating and rapidly disseminating evidence regarding e-cigarette safety and efficacy for smoking cessation. Without such efforts, PCPs will continue to devise their own beliefs and practices regarding e-cigarettes that are likely to be difficult to change once established [27].

## Figures and Tables

**Table 1:** Semi-structured interview questions with main probes used.

How do you typically ask your patients about their tobacco use status? How do you go about counselling patients who are current tobacco users? Have you ever asked any of your patients about their e-cigarette use? If yes, How did you go about doing that? Have any of your patients ever asked you about e-cigarettes? If yes, Can you estimate how often over the past year? AND Can you tell me a typical question patients asked? Do you know if any of your patients use e-cigarettes? If yes, What are your thoughts about that? Did you recommend e-cigarettes to any of your patients? If yes ➔ continue probing Was there something specific about the patient that led you to recommend/NOT recommend it? What was it about the patient? Something they said? What are your thoughts regarding e-cigarettes and other modes of tobacco use? > How do you think e-cigarettes compare to other available tobacco products? What are your thoughts regarding e-cigarettes and smoking cessation? > How do you think e-cigarettes compare to other cessation aids available? Are there specific patients to whom you might be more or less likely to recommend e-cigarettes? > Give me an example of a patient to whom you are most likely/least likely to recommend e-cigarettes.

## Abbreviations

The following abbreviations are used in this manuscript:

U.S.	United States
PCP	Primary Care Physician
FDA	United States Food and Drug Administration
ACORN	Ambulatory Care Outcomes Research Network
